# Branch retinal vein occlusion in a case of recalcitrant diffuse anterior scleritis treated with tofacitinib

**DOI:** 10.1186/s12348-023-00359-w

**Published:** 2023-09-27

**Authors:** Anitha Manoharan, Harshita Atmakur, Parthopratim Dutta Majumder, Jyotirmay Biswas

**Affiliations:** 1 Department of Uvea, Sankara Nethralaya, 41, College Road, Nungambakkam, Chennai 600006 India; 2 Director of Uveitis and Ocular Pathology Department, Sankara Nethralaya, 41, College Road, Nungambakkam, Chennai, 600006 India

**Keywords:** Recalcitrant scleritis, JAK/STAT inhibitors, Non-necrotizing scleritis, Tofacitinib

## Abstract

A 47-year-old woman with hypertension and rheumatoid arthritis presented with non-necrotizing scleritis in both eyes. Despite a course of oral corticosteroids, she continued to experience persistent symptoms. A rheumatologist was consulted and initiated treatment with tofacitinib, a JAK/STAT inhibitor. Treatment with tofacitinib and oral corticosteroids resulted in an improvement in the scleritis in both eyes. However, a fundus examination of her left eye revealed a superior-temporal branch retinal vein occlusion. Given the growing concern regarding the increased risk of thromboembolic events with tofacitinib therapy, it is essential to consider the risk of retinal vascular occlusions when starting tofacitinib therapy, particularly in patients with underlying systemic comorbidities.

## Introduction

The treatment of non-infectious scleritis involves the use of corticosteroids and immunosuppressive agents in a stepwise manner. The severity of the scleral inflammation, response to the treatment, and the underlying cause guide the treatment regime in these patients. Despite conventional therapies, some cases of non-infectious scleritis remain refractory, leading to an unmet need for alternative treatment options. In recent years, biological agents have shown promise in the management of non-infectious scleritis. These agents target specific pathways or components of the immune system and have been shown to be effective in reducing inflammation. Additionally, a new class of immunomodulatory agents that inhibit *Janus kinase* signal transducer and activator of transcription (JAK-STAT), an intracellular signaling pathway, has been introduced in the management of various systemic rheumatic diseases. JAK inhibitors have shown efficacy in the treatment of various autoimmune and inflammatory disorders, including rheumatoid arthritis, psoriasis, and inflammatory bowel disease. Tofacitinib, a JAK inhibitor, has been recently employed in the management of treatment-resistant cases of uveitis and scleritis. While there are reports of thromboembolic events associated with the use of tofacitinib, no cases of retinal vascular occlusion suggesting a possibility of thromboembolic episodes inside the eye have been reported. We present a case of branch retinal vein occlusion (BRVO) in a patient with diffuse non-infectious non-necrotizing scleritis who was treated with tofacitinib. Tofacitinib, a JAK inhibitor, has recently been used to treat treatment-resistant cases of uveitis and scleritis. While thromboembolic events have been reported with its use, there have been no reports of tofacitinib-associated retinal vascular occlusion, which suggests the possibility of thromboembolic episodes occurring inside the eye.

## Case report

A 47-year-old female with a history of hypertension and rheumatoid arthritis presented to our clinic with complaints of ocular pain and redness in both eyes. She was already being treated by a rheumatologist and was taking hydroxychloroquine 400 mg once daily. She had previously sought care elsewhere for her eye problem, where she was diagnosed with scleritis and treated with systemic corticosteroids. Her laboratory investigations, which were conducted elsewhere, revealed a negative Mantoux test, negative antinuclear antibody (ANA) and antineutrophil cytoplasmic antibody (ANCA) results. She was also under the care of an internist at her place of residence, who was monitoring her hypertension with routine laboratory tests and antihypertensive medications.

**Fig. 1:**
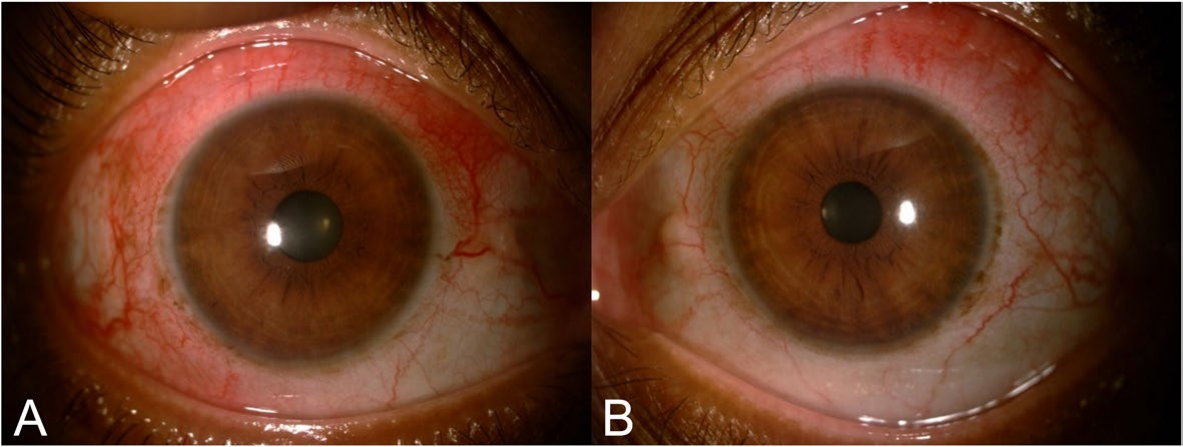
Slit lamp pictures of the right eye (**a**) and the left eye (**b**) showing diffuse deep episcleral congestion, with active non-necrotizing anterior scleritis with a quiet anterior chamber and clear lens

At the presentation to us, her best corrected visual acuity (BCVA) was 6/6 in both eyes, with intraocular pressure measured by applanation tonometry was 10 mm of hg in both eyes. Slit lamp examination of both eyes revealed diffuse deep episcleral congestion and scleral edema, with a quiet anterior chamber (Fig. 1a and b). Anterior segment optical coherence tomography confirmed the findings with an increased scleral thickness corresponding to areas of scleral inflammation in both eyes. Fundus examination of both eyes was unremarkable. A diagnosis of active non-necrotizing anterior scleritis was made, and consultation from a rheumatologist was sought to rule out systemic rheumatic diseases and initiate immunomodulatory therapy.

**Fig. 2:**
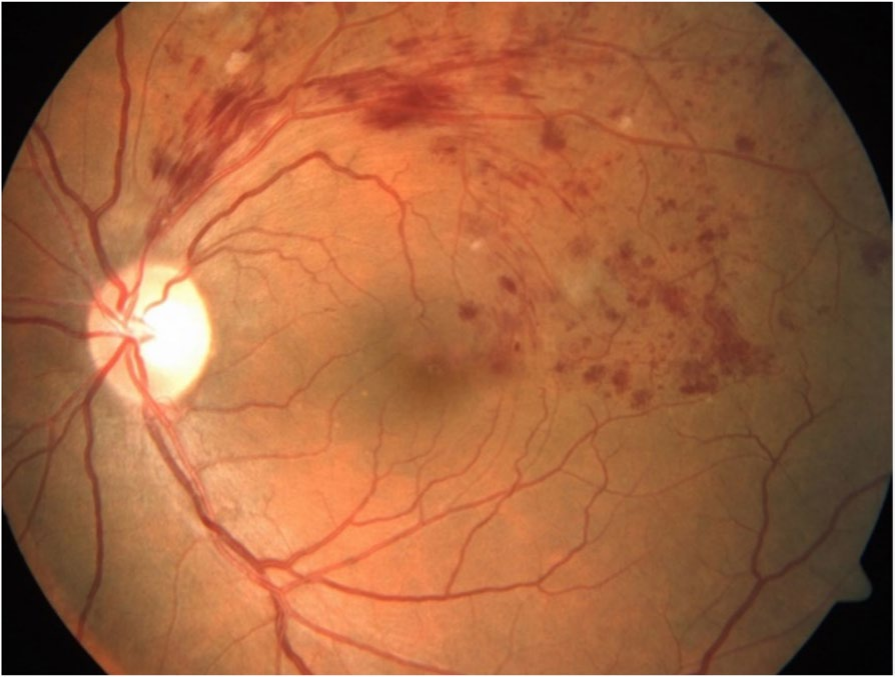
Color fundus photograph of the left eye showing scattered superficial retinal hemorrhages with few cotton wool spots along the superior-temporal arcade with tortuous vessels

The patient was started on oral tofacitinib 5 mg twice daily, along with tapering doses of oral steroids. A follow-up visit after two months revealed complete resolution of scleral inflammation in both eyes. Her BCVA was 6/6 in both eyes, and intraocular pressure was 12 mm of Hg in both eyes. Fundus examination of the left eye revealed scattered superficial retinal haemorrhages with few cotton wool spots along the superior-temporal arcade with tortuous vessels (Fig. 2). Fundus examination of the right eye was unremarkable. Optical coherence tomography was normal in both eyes. No evidence of neovascularization was seen in fundus fluorescence angiography. The patient was advised to stop tofacitinib and was started on mycophenolate mofetil 1 gram twice daily and oral steroids in tapering doses. The patient was advised to come for a review after one month.

## Discussion

Tofacitinib, an oral small-molecule Janus kinase inhibitor, has been reported by several authors, including our group, to be effective in treating scleritis at a dosage of 5 mg twice daily [1–3]. The drug has been approved by US Food and Drug Administration (FDA) for the treatment of rheumatoid arthritis (RA), psoriatic arthritis, and ulcerative colitis. Our patient was started on oral tofacitinib before using conventional immunosuppressives. Several studies have highlighted the effectiveness of tofacitinib as monotherapy, and there has been an increasing trend in the use of the drug in the management of RA in recent years [4]. However, the FDA has issued a warning about the increased risk of blood clots and death in patients with rheumatoid arthritis who were treated with a 10 mg twice-daily dose of tofacitinib. This warning was based on interim data from an ongoing safety study that showed a higher risk of pulmonary embolism and overall mortality in patients receiving the higher dose compared to those receiving a TNF inhibitor [5]. The risk was found to be higher in patients over 50 years old, with a history of cardiovascular disease, or receiving high doses of corticosteroids [6]. Patients with pre-existing thromboembolic risk factors, such as those on prothrombotic or antithrombotic treatments, were also found to be at increased risk of thromboembolic episodes with the use of tofacitinib [7]. Also, long-standing inflammatory systemic disorders, increased age, and traditional cardiovascular risk factors such as obesity, diabetes, hypertension, hyperlipidemia, and smoking, further increased the risk of thrombosis [8]. Other adverse events associated with the use of tofacitinib include increased risk of herpes zoster infection, [9] reactivation of tuberculosis, [10] malignancy, [11] increased levels of liver transaminases, and serum lipid derangements [12]. The index case presented with diffuse active scleral inflammation, along with a history of rheumatoid arthritis and systemic hypertension. Given the severity of the inflammation, its resistance to systemic corticosteroid treatment, and the patient’s underlying systemic disease, both a rheumatologist and ophthalmologist decided to begin tofacitinib therapy. Tofacitinib has been found to be helpful in the management of treatment-resistant cases of scleritis [1–3]. At our setup, the drug is often preferred over the TNF-alpha agents, particularly by the patients, as it is cheaper and available as oral formulation. The tofacitinib therapy was successful in resolving the scleral inflammation, but unfortunately, the patient developed superior-temporal BRVO. It is difficult to determine the exact cause of this event, particularly given the presence of systemic hypertension in the patient. However, it is worth noting that the patient was receiving regular medical care and was closely monitored by an internist. Nevertheless, the potential risk of ocular thromboembolic events must be considered when initiating tofacitinib therapy, particularly in patients with systemic comorbidities.

## Data Availability

Not relevant.
